# Hippocampal microRNA-26a-3p deficit contributes to neuroinflammation and behavioral disorders via p38 MAPK signaling pathway in rats

**DOI:** 10.1186/s12974-022-02645-1

**Published:** 2022-11-24

**Authors:** Changmin Wang, Ye Li, Yuhang Yi, Guiyu Liu, Ruojing Guo, Liyan Wang, Tian Lan, Wenjing Wang, Xiao Chen, Shihong Chen, Shu Yan Yu

**Affiliations:** 1 Department of Physiology, Shandong University, School of Basic Medical Sciences, 44 Wenhuaxilu Road, Jinan, 250012 Shandong People’s Republic of China; 2 Morphological Experimental Center, Shandong University, School of Basic Medical Sciences, 44 Wenhuaxilu Road, Jinan, 250012 Shandong People’s Republic of China; 3 Department of Endocrinology, The Second Hospital, Cheeloo College of Medicine, Shandong University, 247 Beiyuan Street, Jinan, 250033 Shandong People’s Republic of China; 4 Shandong Provincial Key Laboratory of Mental Disorders, School of Basic Medical Sciences, 44 Wenhuaxilu Road, Jinan, 250012 Shandong People’s Republic of China

**Keywords:** Neuroinflammation, Neuroplasticity, MicroRNA-26a-3p, p38 MAPK, Behavioral disorder

## Abstract

**Background:**

Neuronal injury is considered a critical risk factor in the pathogenesis of most neurological and neuropsychiatric diseases. However, the underlying molecular mechanisms and identification of potential therapeutic targets for preventing neuronal injury associated with brain function remain largely uncharacterized. Therefore, identifying neural mechanisms would put new insights into the progression of this condition and provide novel therapeutic strategies for the treatment of these diseases.

**Methods:**

Stereotactic injection of AAV virus was used to knock-down the miR-26a-3p within hippocampus of rats. Behavioral changes was detected by open field test (OFT), elevated plus maze (EPM), forced swim test (FST) and sucrose preference test (SPT). The inflammatory cytokines and related proteins were verified by real-time quantitative PCR, immunoblotting or immunofluorescence assay. Golgi staining and electron microscopy analysis was used to observe the dendritic spine, synapse and ultrastructural pathology. SB203580 (0.5 mg/kg) were administered daily to prevent p38 MAPK via an intraperitoneal (i.p.) injection. Finally, electrophysiological method was used to examine the synaptic transmission via whole-cell patch-clamp recording.

**Results:**

Here, we showed that miR-26a-3p deficiency within hippocampal regions leads to the activation of microglia, increased level of pro-inflammatory cytokines and behavioral disorders in rats, effects which appear to be mediated by directly targeting the p38 mitogen-activated protein kinase (MAPK)–NF-κB signaling pathway. Specifically, we found that the enhanced glia-activation may consequently result in neuronal deterioration that mainly presented as the dysregulation of structural and functional plasticity in hippocampal neurons. In contrast, preventing p38 pathway by SB203580 significantly ameliorated abnormal behavioral phenotypes and neuronal jury resulting from miR-26a-3p knock-down.

**Conclusion:**

These results suggest that the normal expression of miR-26a-3p exerts neuroprotective effects via suppressing neural abnormality and maintaining neuroplasticity to against behavioral disorders in rats. These effects appear to involve a down-regulation of p38 MAPK-NF-κB signaling within the hippocampal region. Taken together, these findings provide evidence that miR-26a-3p can function as a critical factor in regulating neural activity and suggest that the maintaining of normal structure and function of neurons might be a potential therapeutic strategy in the treatment of neurological disorders.

**Graphical Abstract:**

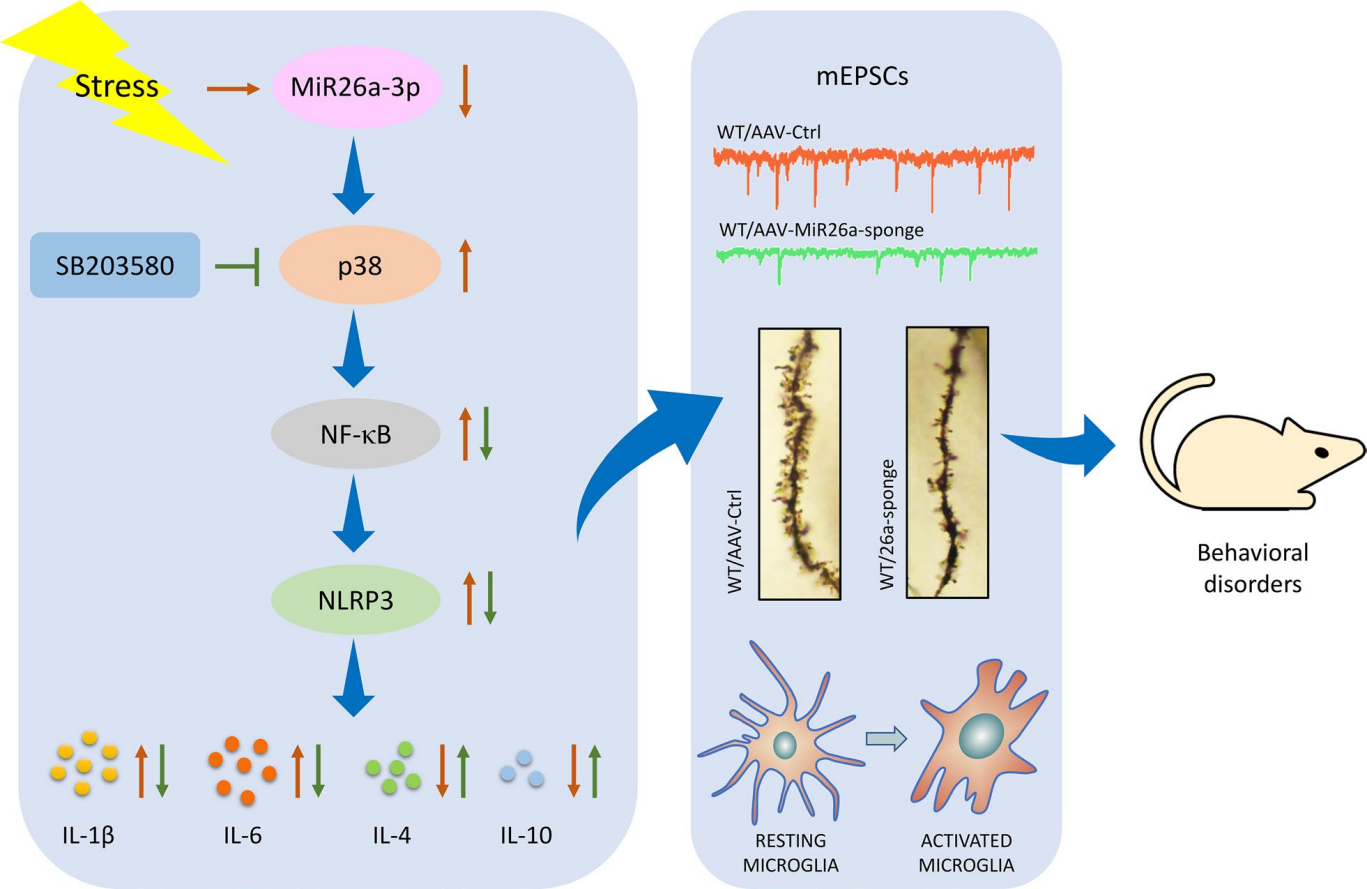

**Supplementary Information:**

The online version contains supplementary material available at 10.1186/s12974-022-02645-1.

## Highlights

1. MiR-26a-3p is down-regulated in several neurological disease-related model.
2. Knock-down of miR-26a-3p within hippocampus induced behavioral disorders in rats.
3. Knock-down of miR-26a-3p promoted neuroinflammation in hippocampus.
4. Knock-down of miR-26a-3p resulted in dysregulation of synaptic plasticity.
5. MiR-26a-3p regulates neural inflammation and plasticity through the p38/NF-κB pathway.

**Supplementary Information:**

The online version contains supplementary material available at 10.1186/s12974-022-02645-1.

## Background

Neuronal injury is considered critically related to the neuronal dysfunction in specific brain regions which thus promotes the progression of neuropsychiatric diseases [1, 2]. Results from our previous studies have demonstrated that increased neuro-inflammatory responses appeared to result in neuronal apoptosis and aggravate dendritic spine impairments, while suppression of inflammation promotes recovery from synaptic injury and depression-like behaviors in animal models of depression [3, 4]. However, the potential for development of corresponding therapeutic treatment targeting these mechanisms associated with abnormal neuronal activity in depression remains largely unknown.

Recent studies have found that external stress can cause epigenetic changes in some genes in brain, and then regulate neuroplasticity and neuronal function [5–7]. MicroRNA (miRNA) is a kind of small RNAs encoded by endogenous genes, about 20–24 nucleotides in length, which play a variety of important regulatory effects on the gene transcription and translation process in cells [8, 9]. Previous studies have reported that miR-26a is highly specifically expressed in brain [10], and can promote the growth of neurite [11], suggesting that miR-26a may play an important role in the neural development and the regulation of neural plasticity. Recent studies found that miR-26a may play a key role in the maintenance of long-term potential (LTP) of synaptic transmission under physiological conditions via regulating the morphological structure and function of neurons [12], and dysregulation of miR-26a is reported to involve in the pathogenesis and progression of many neurological diseases [13–16]. Moreover, some studies have suggested that serum miR-26a is candidate for an auxiliary biomarker for the diagnosis of Alzheimer's disease (AD) [17, 18]. Clinical studies have also found that the expression level of miR-26a in peripheral serum of patients is decreased in major depression, while the antidepressant treatment can increase the expression level of miR-26a [19, 20]. Our recent studies also showed that upregulation of miR-26a-3p rescues hippocampal neuronal anomalies and behavioral disorder in depression animal model [21]. These results suggested that maintaining the expression level of miR-26a may be important to the regulation of neuroplasticity and even the pathogenesis of neuropsychiatric diseases. However, one microRNA usually targets many downstream mRNAs to regulate the translation of corresponding proteins, therefore the detailed molecular pathway through which miR-26a deficits in specific brain region affects the neuronal function and then leads to behavioral abnormalities is not fully understood.

In the present study, we attempted to investigate some of the underlying mechanisms through which miR-26a-3p exerts its neuroprotective effects to prevent behavioral disorders in rats. We found that phosphorylation levels of p38 MAPK were upregulated within the hippocampus, one of the key brain regions associated with psychiatric disorders [2, 22, 23], after knock-down miR-26a-3p. The protein–protein interaction (PPI) network analysis showed that the eukaryotic transcription factor NF-κB interacted with the p38 protein, while the key mediator of immunity, NF-κB, also plays a critical role for the priming of the NLRP3 inflammasome [24]. In contrast, inhibiting p38 MAPK signaling greatly ameliorated the display of depression-like and anxiety-like behaviors caused by miR-26a-3p deficits. The abnormal effects induced by miR-26a-3p deficits appear to be attributable to the exacerbation of neural-inflammatory response and dysregulation of synaptic transmission processes mediated by the p38 signaling pathway. Such findings provided novel insights into the neuroprotective mechanisms targeting miR-26a-3p/p38 pathway that may serve as a potential therapeutic strategy for behavioral disorders in neuropsychiatric diseases.

## Methods

### Animals

Male Wistar rats (weighing 180–190 g) were obtained from Jinan Peng-yue Experimental Animal Breeding Co., Ltd. Male C57/BL 6J mice (weighing 25–30 g) were obtained from Beijing Vital River Laboratory Animal Technology Co., Ltd. All experimental procedures were approved by the Ethics Committee of Shandong University (ECSBMSSDU2020-2-017), and in compliance with the international guidelines for animal research formulated by the Council of International Medical Organizations. The rats and mice were both housed under standard laboratory conditions for one week before experimental procedures, with free access to food and water. All efforts were made to reduce the animals’ suffering in the experiments.

### Regents and antibodies

SB203580 (0.5 mg/kg) was purchased from MedChemExpress Co., Ltd. (USA). Streptozocin (STZ) and 0.1 M citrate buffer (pH 4.5) were purchased from Solarbio Co., Ltd. Lipopolysaccharide (LPS) and dimethyl sulfoxide (DMSO) were purchased from Sigma-Aldrich Co. (St Louis, MO, USA). The polyclonal rabbit anti-CD11b (ab184307) and polyclonal rabbit anti-CD45 (ab10558) were purchased from Abcam Co. (Cambridge, UK). The polyclonal rabbit anti-NF-κB p65 (BS90940) was purchased from BioWorld Technology, Inc. The polyclonal rabbit anti-GAPDH (10494-1-AP) purchased from Proteintech Technology and the polyclonal rabbit anti-P38 (9212s) was purchased from Cell Signaling Technology. Constructed adeno-associated virus (AAV9-CMV-eGFP-miR-26a-3p-sponge vector) was purchased from Gene-Chem Co. (Shanghai, China).

### Chronic unpredicted mild stress (CUMS) model

The rats were acclimated in the experimental animal room for a week. In brief, the rats were individually housed for 5 weeks. These rats were subjected to chronic stressors including clipping tails (2 min), cold swimming (5 min, 4 ℃), cage shaking (5 min), physical restraints (2 h), food and water deprivation (24 h), wet bedding (24 h), cage tilting (24 h), overnight illumination. Each rat was given one stimulation in a random order daily [25].

### LPS-induced depression model

The rats received intraperitoneal (i.p.) injections of LPS (0.5 mg/kg) once a day and the process lasted for 10 days. LPS was prepared freshly prior to injection. Rats in the control group were injected with the same dose of normal saline every day [26].

### Diabetic encephalopathy model

STZ was freshly prepared in cold 0.1 M citrate buffer (pH 4.5) and then injected intraperitoneally into rats using a single dose of 60 mg/kg body weight, and the feeding was continued for 12 weeks to develop diabetic encephalopathy (DE) model [27].

### Acute restraint stress mice model

Acute restraint stress (ARS) was used to induce disordered behaviors in mice. Male C57/BL 6 J-wild-type mice (8 weeks) were immobilized for 5 h using an individual retainer to limit all physical movement but causing minimum pain to them. Mice were deprived of water and food during the ARS experiment [28].

### Drug treatments

SB203580 (0.5 mg/kg) or DMSO (0.1%) were administered via an intraperitoneal injection for two weeks after viral injection. LPS (0.5 mg/kg) was dissolved in 0.9% saline at the concentration of 10 mg/ml before injection. The dose and route of SB203580 administration is based upon previous study with minor modifications [29].

### Behavioral tests

All behavioral tests of rats were conducted during the dark circadian period (19:00–24:00). The tests and analysis were performed by an experimenter blind to the treatment group.

### Novel object recognition test

The novel object recognition (NOR) test is a widely used behavioral test to assess hippocampus-dependent recognition memory. The experiment was based on previous research with minor modifications [30]. To test recognition, rats were placed in an empty box (100 × 100 × 50 cm) for habituation 1 d before the test. On the first day of the test (familiarization phase), the rats received 5 min to explore the two identical objects in the recognition box. On the second day (24 h later, test phase), the researcher returned the rats to the open-field recognition box with two objects, the initial object explored during the familiarization phase and a newly introduced novel object. In this phase, each animal received 5 min to explore the two objects freely. A video system was used to record the rats' movements in the open field, and researchers measured the amount of time the rats spent in contacting with new and familiar objects by watching videos. The discrimination index was calculated according to the following formula: discrimination index (time spent on novel objects/total time spent on both exploring objects) × 100%. Exploration of an object was defined as the animal placing its nose within 2 cm of the zone where the object located.

### Sucrose preference test

Sucrose preference test (SPT) was used as the classical method to define anhedonia [31]. Individually rat from different groups was given two water bottles during the test period. On the first day, two bottles were both filled with tap-water. Twenty-four hours later, one bottle was filled with tap-water and the other one was replaced with 2% sucrose solution. The two water bottles position were changed every twelve hours (right one to left, left one to right) to ensure that the rats did not have a side preference. On the third day, rats were deprived of water and food for 24 h. In the next day, two water bottles (one bottle was filled with tap-water and the other one was 2% sucrose solution) were placed in each cage. The position of the bottles was switched after one hour during the test to prevent the possible effects of a side preference. The amount of liquid remaining in bottles was measured to calculate the liquid consumption. The calculation formula of sucrose preference (%) is as follows: sucrose consumption/(sucrose consumption + water consumption).

### Forced swimming test

Forced swimming test (FST) was used to define behavioral despair [32]. In the first training phase, rats were placed individually in a plastic bucket (80 cm high, 30 cm diameter) filled with water 50 cm high at suitable temperature for 15 min of forced swimming. The testing phase was twenty-four hours after training. The rats were orderly placed in the bucket for 5 min. The immobility time and swimming time were recorded during the test. The new water replaced dirty water before the next rat swimming test. The rats cannot touch the bucket bottom or escape in test period.

### Open field test

Open field test (OFT) was used to assess basic activities and anxiety-like behavior [33]. The open field is a black wooden open field box (100 × 100 × 50 cm). The inner side surface was cleaned with 75% ethanol to be free of all dirt. The rats were individually placed in the center of the open field and allowed to move freely for five minutes. The video tracking software (SMART 2.5, Panlab Harvard Apparatus, Spain) was used to record the activities of rats. The total distance of horizontal locomotion and the time spent in the central area were analyzed to assess anxiety-like behavior.

### The elevated plus maze test

The elevated plus maze (EPM) was used to assess anxiety in rats [33]. The EPM consists of two closed arms (50 cm × 10 cm), two open arms (50 cm × 10 cm) and a central platform (10 cm × 10 cm) at the intersection of the arms. The animal was firstly placed on the central platform with their heads facing to the open arms during 5-min test period. Time spent in the open arms and the number of entries into open arms in 5 min were recorded by video tracking software (SMART 2.5, Panlab Harvard Apparatus, Spain).

### Tail suspension test

The experiment was based on previous research with minor modifications [34]. The rat's tail was cleaned and taped to the hook of the tail hanging device. Rats were suspended 40 cm above the floor for 5 min. The immobility time was recorded during the test. Immobility was defined as a lack of attempt to move their limbs and staying in the vertical posture during suspension. At the end of each rat's experiment, the device was cleaned with 75% alcohol.

### Virus injections

Rats were deeply anesthetized and placed in a stereotaxic apparatus (Stoelting, USA). The AAV9-CMV-eGFP-miR-26a-3p-sponge vector virus, which acts as a sponge to inhibit the functions of endogenous miR-26a-3p, was bilaterally injected into the hippocampus region (bregma: − 3.24 mm; medial/lateral: ± 1.5 mm; dorsal/ventral: − 4.5 mm) with use of the electric microinjection pump (Stoelting, USA) at the speed of 0.11 µl/min. After the required volume has been injected, the micro syringe remained in the place for at least 5 min and was slowly withdrawn from the tissue. Rats were placed in warm conditions to resuscitate slowly and rest for three days for the following study. All injection sites were verified by immunofluorescence slice before further experiments and only rats of correct injection site were used for analysis.

### Immunofluorescence staining

After all behavioral tests, the rats were anesthetized and then perfused with heparin sodium saline and 4% paraformaldehyde (PFA). Brains of rats were fixed in PFA overnight followed by a graded dehydration (10%, 20% and 30% sucrose solution) at 4 °C and then were made into slices (30 μm) using the frozen slicer. The slices were washed 3 × 5 min in 1 × PBS and incubated in the blocking solution (1 × PBS + 5% goat serum + 2.5% BSA + 0.2% Triton X-100) for 1 h. Slices were incubated with primary antibodies in the blocking solution at 4 °C overnight. On the second day, slices were incubated with matched secondary antibody (1:1000, Invitrogen) in the blocking solution at 4 °C for 1 h and 5 min in DAPI (Beyotime Biotechnology C1002). Images were obtained from high-speed confocal platform (Dragonfly 200).

### Transmission electron microscopy (TEM)

Fresh sample of hippocampal tissue (size: 1 mm × 1 mm × 1 mm) was dissected and placed in 2.5% glutaraldehyde at room temperature for 0.5 h and then at 4 °C overnight. The samples were washed 3 × 10 min in 0.1 M PBS (pH 7.4) and then fixed with 1% osmium for 1.5 h. After that, samples were washed 3 × 10 min in ultrapure water followed by dehydration and infiltration. Ultrathin section (Leica UC7) of tissues were performed after embedding in resin. Pictures were finally captured by Talos F200C TEM (JEM-1200EX TEM) and randomly selected from each rat for analysis.

### Western blot analysis

Rats were anesthetized with sodium pentobarbital (150 mg/kg, i.p.) and the hippocampus regions were carefully dissected for western blot analysis at 24 h after behavioral tests. Briefly, hippocampus was homogenized in ice-cold RIPA buffer (catalog R0020, Solarbio) with a cocktail of protease/phosphatase inhibitors (catalog P1260, Solarbio). The homogenate was centrifuged at 12,000×*g* for 25 min at 4 °C, and supernatants were collected. Protein concentrations in hippocampus was determined using BCA Protein Assay Kits (catalog CW0014s, CWBIO). Equal amounts of protein samples (30 μg) were separated by SDS-PAGE and transferred onto PVDF membranes, which were blocked in 5% nonfat milk for 1 h, and then incubated overnight at 4 °C with the appropriate primary antibodies including polyclonal rabbit anti-CD11b (1:1000, catalog ab184307, Abcam), polyclonal rabbit anti-NF-κB p65 (1:1000, catalog BS90940, BioWorld), monoclonal rabbit anti-NLRP3 (1:1000, catalog ab263899, Abcam), polyclonal rabbit anti-GAPDH (1:5000, catalog 10494-1-AP, Proteintech), polyclonal rabbit anti-CD45 (1:500, catalog ab10558, Abcam) and polyclonal rabbit anti-P38 (1:1000, catalog 9212s, Cell Signaling Technology). The membranes were incubated with secondary horseradish peroxidase-conjugated antibodies goat anti-rabbit IgG (1:5000, catalog ZB-2301, Zhongshan Golden Bridge Biotechnology) and Peroxidase-conjugated goat anti-mouse (1:2000, catalog ZB-2305, Zhongshan Golden Bridge Biotechnology). Blots were detected using an enhanced chemiluminescence kit (catalog E412-01, Vazyme). Protein band densities were quantified using Image-J software and the experiment with the samples of each rat was replicated at least three times.

### Reverse transcription PCR (RT-PCR) and real-time quantitative PCR

Total RNA was extracted from tissues of hippocampus using the RNA extraction kit (catalog AP-MN-MS-RNA-50, Axygen) according to the manufacturer’s instructions.

### For mRNA

Reverse transcribe RNA into cDNA using SureScript™ First-Strand cDNA Synthesis Kit (catalogQP056, GeneCopoeia™). The reverse transcription reaction system was prepared using the following components: 1 µl SureScript RTase Mix (20x), 4 µl SureScript RT Reaction Buffer (5x), 1ug or 10ug total RNA. The whole system was replenished to 20 µl with ddH_2_O (RNase/DNase free). Expression levels of mRNA were determined using BlazeTaq™ SYBR Green qPCR Mix 2.0 (catalog QP031, GeneCopoeia™). QPCR reaction system was prepared using the following components: 2 µl 5xBlazeTaq qPCR Mix, 2 µl specific primers, 1-2 µl cDNA (diluted 1:20), the whole system was replenished to 10 µl with ddH_2_O (RNase/DNase free).

### For microRNA

Reverse transcribe RNA into cDNA using All-in-OneTM miRNA qPCR Detection Kit 2.0 (catalog QP115, GeneCopoeia™). The reverse transcription reaction system was prepared using the following components: 1 µl Poly-A Polymerase, 1 µl SureScript™ RTase Mix (20x), 4 µl 5xPAP/RT Buffer, 100 ng–1ug Total RNA. The whole system was replenished to 20 µl with ddH_2_O (RNase/DNase free). The reverse transcription process was performed on PCR thermal cycler (Hangzhou Jingle Scientific Instrument Co, Ltd). Expression levels of miRNA were determined using All-in-One™ miRNA qPCR Detection Kit 2.0 (catalog QP115, GeneCopoeia™). QPCR reaction system was prepared using the following components: 5 µl 2xAll-in One TM qPCR MIX, 1 µl Universal Adaptor PCR Primer (diluted 1:24), 1 µl specific primer, 1–2 µl cDNA (diluted 1:7.5). The whole system was replenished to 10 µl with ddH_2_O (RNase/DNase free.

Real-time quantitative PCR analysis was performed on a Bio-Rad iCycler system (Bio-Rad). GAPDH served as a loading control for the sample to test for mRNA and Rno-U6 served as a loading control for the sample to test for miRNA. The mRNA expression levels and miRNA expression levels were evaluated using the 2 − (ΔΔCt) method. All samples were repeated twice to reduce the error and all the special primers are obtained from BGI Genomics Co., Ltd (Additional file 1: Table S1).

### Golgi staining

Golgi staining was performed as described previously [35]. Fresh brain tissues of rats were collected for the Golgi–Cox staining test according to the user manual provided by the FD Rapid Golgi Stain™ kit (PK401, FD Neuro Technologies. INC). Brains were sectioned into 150-μm coronal slices with the vibratome (Leica VT1200S, Germany), and collected on the pre-gelatin-coated microscope slides, dehydrated with a gradient series of alcohols, finally cleared in xylene and covered slip. Slides were kept at room temperature, away from light. Stained pyramidal neurons in hippocampal region were captured with use of panoramic digital section scanning microscope (Olympus microscope VS120, Japan). Dendritic spine densities were calculated as the number of dendritic spines per 10 μm of dendrite length. All images were Fiji (Image J, NIH) processed, including Sholl analysis.

### Molecular docking

The protein structures of p38 and p65 derived from rat and human were obtained, respectively, from UniProt database (https://www.uniprot.org/), which is a database of sequence and function information for protein where a large amount of protein information derived from the research literature. Gramm-x server (http://vakser.bioinformatics.ku.edu/resources/gramm/grammx) was used for rigid molecular docking between proteins to evaluate the possibility of their interaction. P38 is defined as a ligand while P65 as a receptor. Among the output models, the first one was used as the final model and then PyMOL and LigPlot + were used for visualization.

### Electrophysiology recording

Hippocampal slice preparations and electrophysiological recordings were performed according to procedures described previously [21]. Animals were decapitated and brains were removed transferred to the ice immediately. All brain slices (300 mm) were prepared with use of the microtome (VT1200s, Leica, Germany) at rate of 0.18 mm/s velocity in 1 × cutting solution (119 mM choline chloride, 30 mM glucose, 26 mM NaHCO_3_, 7 mM MgSO_4_, 2.5 mM KCl, 1 mM NaH_2_PO_4_, 1 mM CaCl_2_, 3 mM sodium pyruvate, 1.3 mM sodium l-ascorbate and 1 mM kynurenic acid). Brain slices were maintained for 30 min at 33 ℃ in the recovery solution containing 8 mM NaCl, 24 mM NaHCO_3_, 4 mM MgCl_2_, 2.5 mM KCl, 1.2 mM NaH_2_PO_4_, 0.5 mM CaCl_2_, 25 mM glucoses and 50 mM sucrose, and then at room temperature for at least 30 min. All solutions during the experiments were continuously infused with 95% O_2_/5% CO_2_. Whole cell recordings were performed by use of MultiClamp 700B amplifier and the glass pipettes (4–6 MΩ, World Precision Instruments) filled with the internal solution (115 mM CsMeSO_3_, 20 mM CsCl, 10 mM HEPES, 2.5 mM MgCl_2_, 4 mM Na_2_-ATP, 0.4 mM Na-GTP, 10 mM Na-phosphocreatine, and 0.6 mM EGTA). For mEPSC recordings, neurons of rats were clamped at − 70 mV in the presence of TTX (1 μM) and picrotoxin (100 μM) in the ACSF (artificial cerebral spinal fluid: 120 mM NaCl, 3.5 mM KCl, 2.5 mM CaCl_2_.2H_2_O, 1.3 mM MgSO_4_, 1.25 mM NaH_2_PO_4_, 26 mM NaHCO_3_, 10 mM glucose). Data were filtered at 2 kHz and sampled at 10 kHz using Digidata 1440A. For sEPSC recordings, neurons were recorded at − 70 mV in the presence of picrotoxin (100 μM) in the ASCF. Data were analyzed with Mini Analysis Program (Mini 60, Synaptosoft).

### Statistical analysis

All data were present as the means ± SEMs, and statistical analyses were performed using GraphPad Prism software version 8.0. Independent group Student’s *t*-tests (two-tailed) were used to analyze in comparisons between two independent groups. One-way or two-way analysis of variance (ANOVA) was used to analyze in comparisons between three or more groups followed by Tukey’s post hoc test. All experiments were repeated at least three times comprising a minimum of 6 animals/group. *P* value < 0.05 was required for results to be considered statistically significant.

## Results

### MiR-26a-3p deficiency in hippocampus caused behavioral disorders in rats

**Fig. 1:**
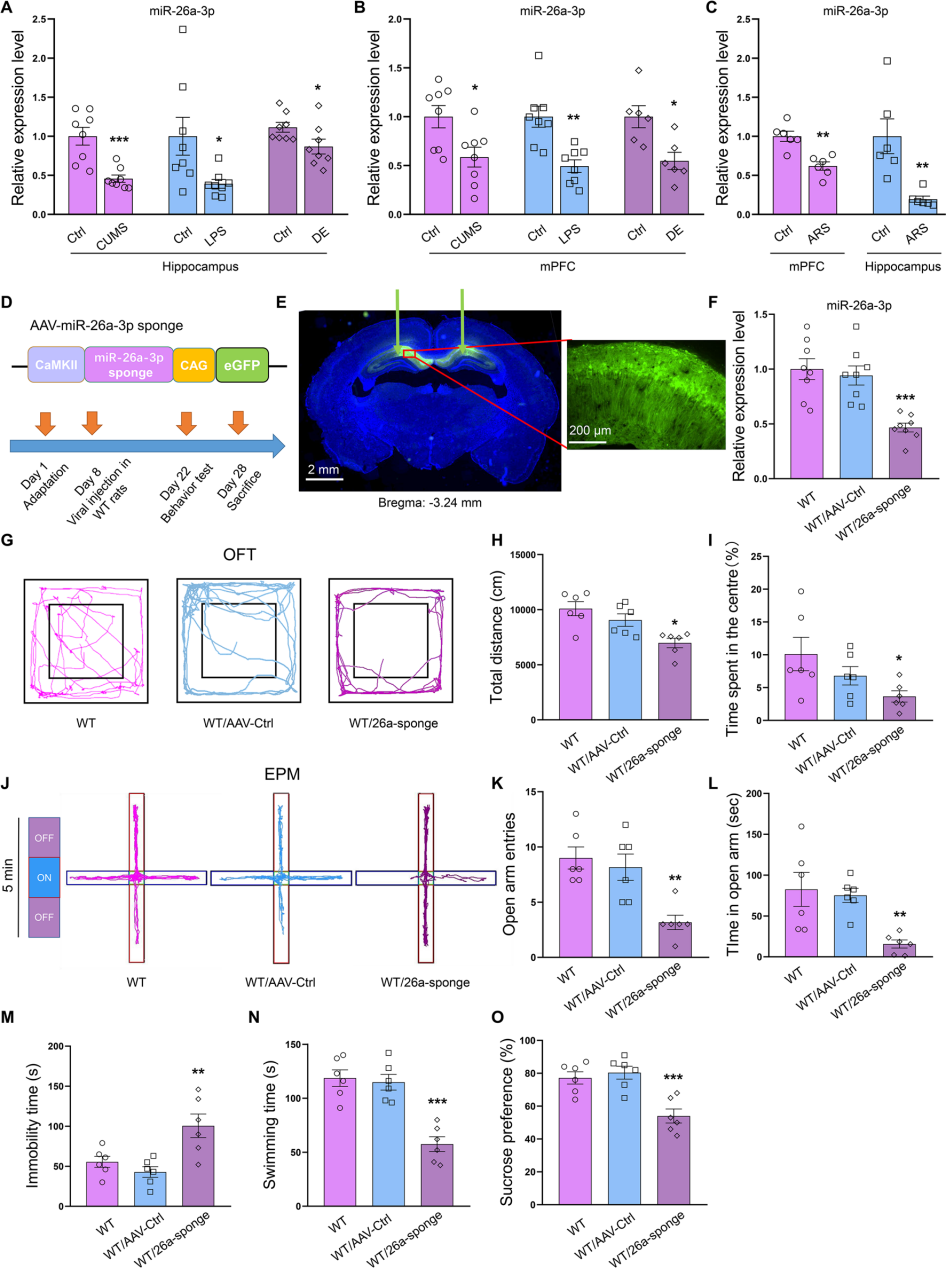
Knock-down of miR-26a-3p in hippocampus caused behavioral disorders in rats. **A** The expression of miR-26a-3p in CA1 hippocampal region in animal models. **B** The expression of miR-26a-3p in DG hippocampal region in animal models. **C** The expression of miR-26a-3p in vmPFC in animal models. **D** Schematics of AAV vectors engineered to knock-down miR-26a-3p and the experimental paradigm. **E** Illustration of AAV viral infusion into the hippocampal region. Scale bar is 2 mm. **F** Expression level of miR-26a-3p in the hippocampus. **G** Motion traces of rats in the open field test (OFT). **H** Total distance in the OFT was decreased after knock-down of miR-26a-3p. **I** Time spent exploring the center area was decreased after knock-down of miR-26a-3p. **J** Motion traces of rats in the elevated plus maze (EPM). **K** The number of entering open arms was decreased in miR-26a-3p knock-down rats. **L** Time spent in the open arms (%) was decreased in miR-26a-3p knock-down rats. **M** The immobility times in forced swim test (FST) were increased in miR-26a-3p knock-down rats. **N** The swimming times in FST was decreased in miR-26a-3p knock-down rats. **O** The percent of sucrose consumption was decreased in sucrose preference test. Data are presented as the means ± SEMs. *N* = 8 per group. NS, not significant (*P* > 0.05); *P < 0.05, **P < 0.01, ***P < 0.001

The expression of miR-26a-3p in the ventromedial prefrontal cortex (vmPFC), hippocampal CA1 and DG regions were examined in several neurological disease-related animal models: chronic or acute stress-treated, LPS-treated and diabetic encephalopathy rats. The qRT-PCR results showed that miR-26a-3p were significantly decreased within these brain regions of these animal models versus normal rats (Fig. 1A, B). The expression of miR-26a-3p in the ventromedial prefrontal cortex (vmPFC) and hippocampal region was also decreased in Acute Restraint Stress mice model (Fig. 1C). To investigate the effects of miR-26a-3p on neuronal function, we constructed AAV-miR-26a-3p-sponge virus to knock-down miR-26a-3p function via bilateral stereotaxic infusion into the hippocampus of normal rats, while a vector included scramble sequence was used as a negative control (Fig. 1D, E). The validation of knock-down efficiency showed a significant decrease in hippocampal regions compared to the control group (*P* < 0.001, Fig. 1F). Results from the open field test showed that the total distance (*P* < 0.05, Fig. 1G, H) and the time spent in the center (%) (*P* < 0.05, Fig. 1I) decreased after knock-down of miR-26a-3p; moreover, the results from elevated plus maze test showed that the number (*P* < 0.01, Fig. 1J, K) of entries into the open arms and the time (*P* < 0.01, Fig. 1L) stayed in the open arms of rats was significantly reduced after knock-down miR-26a-3p, which demonstrated the anxiety-like behavior in rats. In addition, results from the forced swim test demonstrated that knock-down of miR-26a-3p significantly increased immobility times (*P* < 0.001, Fig. 1M) and decreased swimming times (*P* < 0.001, Fig. 1N) in rats as compared to the control group, as well as decreased the sucrose consumption percent as compared with that of the control group (*P* < 0.001, Fig. 1O), which suggested rats showed depression-like behaviors. The findings from these behavioral tests demonstrate the behavioral disorders in miR-26a deficiency rats.

### MiR-26a-3p directly targets the p38 MAPK mRNA to regulate protein translation

**Fig. 2:**
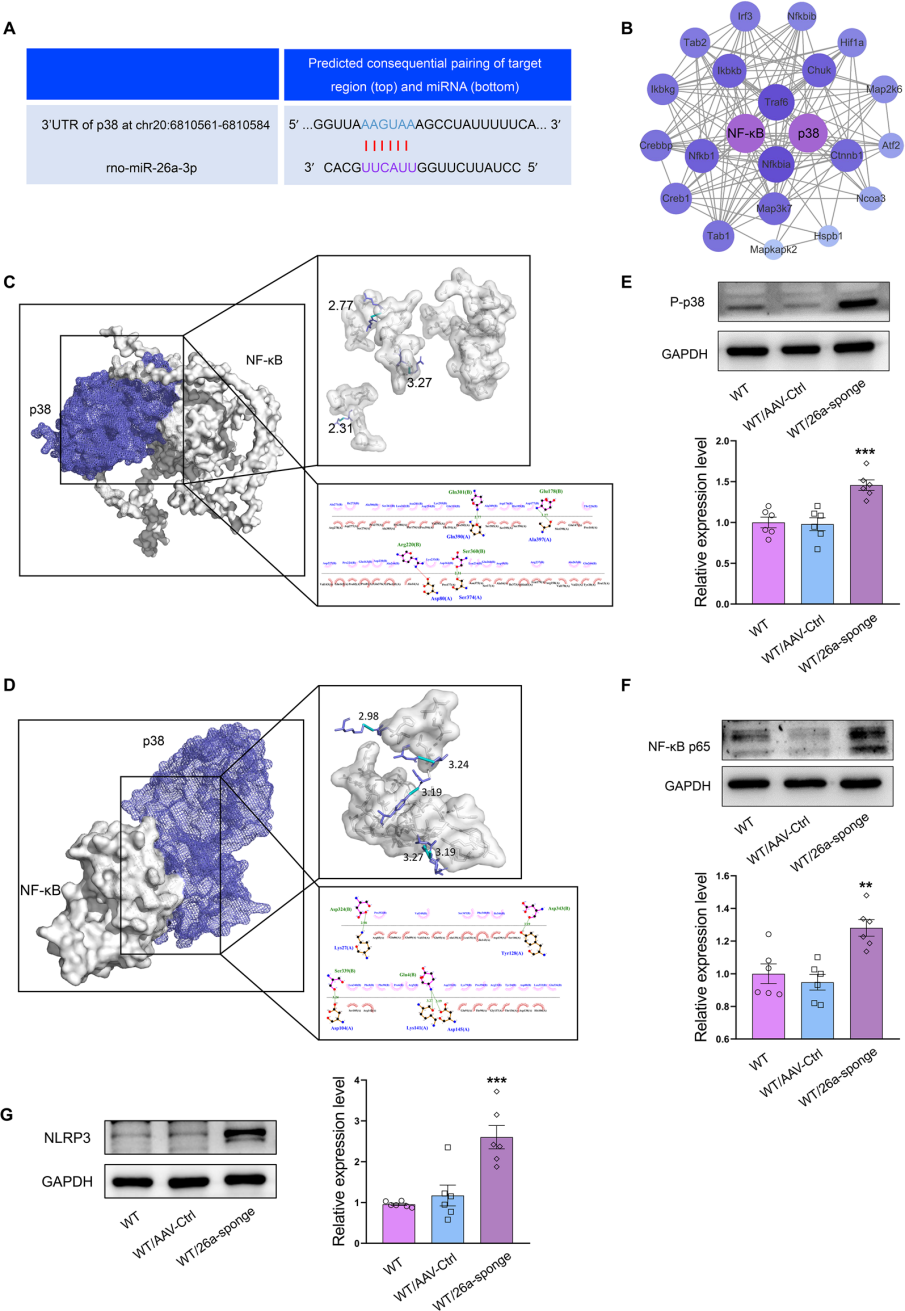
MiR-26a-3p directly targets the p38 MAPK signaling pathway. **A** Predicted putative seed-matching sites between miR-26a-3p and p38. **B** The protein–protein interaction (PPI) network of p38 MAPK and NF-κB. **C** The possible interaction mode of p38 and NF-κB derived from rats. **D** The possible interaction mode of p38 and NF-κB derived from human. **E** Knock-down miR-26a-3p increased the protein expression of p38 MAPK. **F** Knock-down miR-26a-3p increased the phosphorylated level of p65. **G** Knock-down miR-26a-3p increased the expression level of NLRP3. Data are presented as the means ± SEMs. *N* = 6 per group. *P < 0.05, **P < 0.01, ***P < 0.001

We next aimed to identify the potential targets for miR-26a-3p. One miRNA is usually involved with the regulation of multiple target genes and thus associated with multiple signaling pathways. Among the potential genes regulated by miR-26a-3p, it was predicted that the 3′-UTR of p38 MAPK mRNA contains a putative match site for the seed sequence of miR-26a-3p (Fig. 2A). Moreover, the protein–protein interaction (PPI) network analysis showed that there may be exist a direct interaction between the eukaryotic transcription factor NF-κB, which was identified as being associated with inflammation signaling pathways, and the p38 protein (Fig. 2B). The results of molecular docking between proteins suggested the possibility of interaction between p38 and NF-κB derived from rats (Fig. 2C). The left figure of Fig. 2C shows the interaction mode diagram of p38 and NF-κB, and the two figures on the right show the interactions across the interface in 3-directions (3D) and 2D, respectively. The possible interaction mode between these two proteins that derived from human were also evaluated by the same method used as above (Fig. 2D). These analyses indicate that NF-κB may be one of the downstream regulators of p38 MAPK signaling. Western blot analysis showed that knock-down of miR-26a-3p in hippocampus significantly increased protein levels of p38 MAPK (*P* < 0.001, Fig. 2E). Meanwhile, NF-κB and NLRP3 were also significantly activated, the phosphorylated level of p65 and expression level of NLRP3 was upregulated accompanied with the upregulation of p38 after knock-down the miR-26a-3p in hippocampus (*P* < 0.01, Fig. 2F, G). These results suggested that *p38* may act as a target gene of miR-26a-3p and likely regulate the activation of NF-κB/NLRP3 pathway in rats.

### MiR-26a-3p deficiency promoted inflammatory responses in hippocampus in rats

**Fig. 3:**
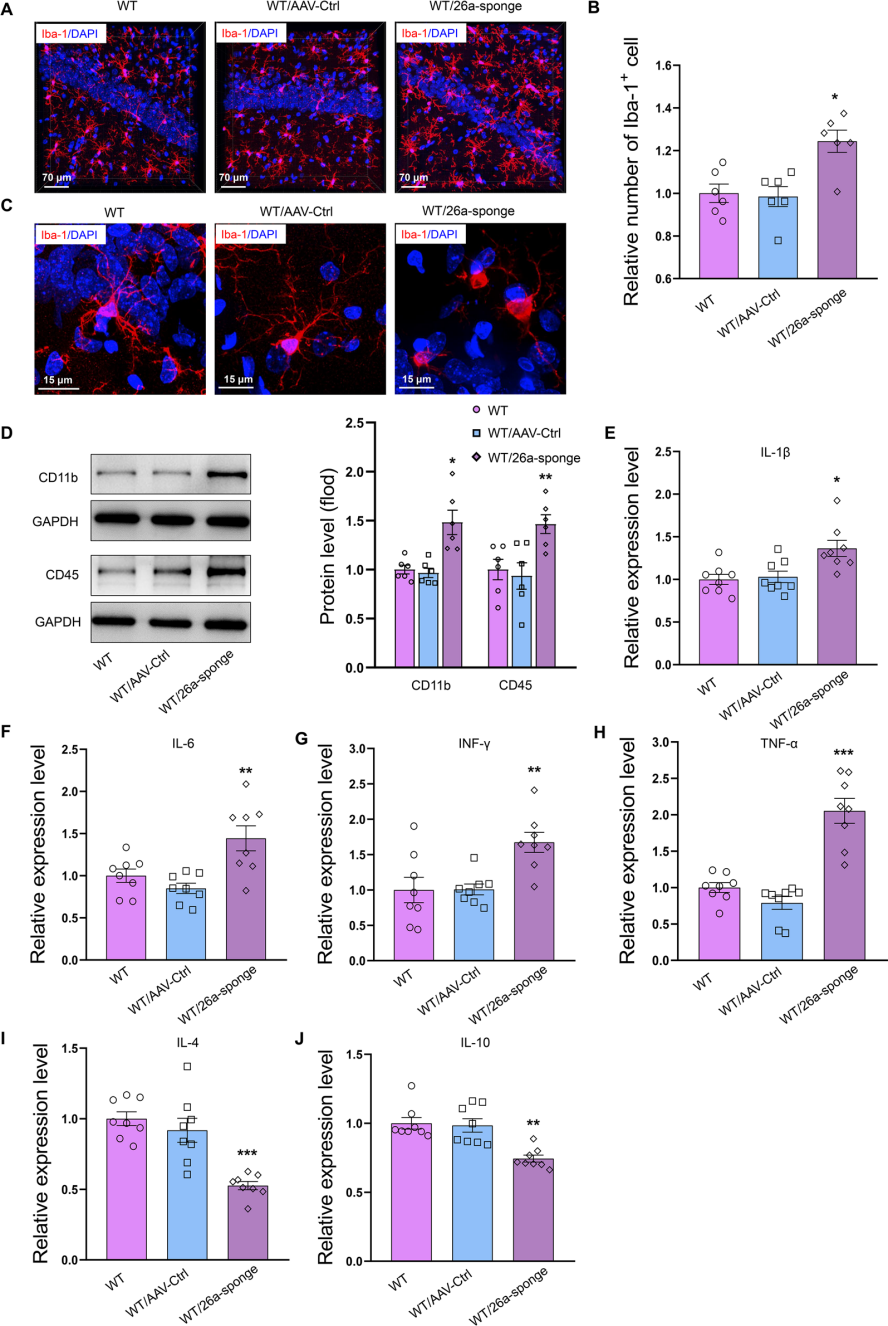
Knock-down miR-26a-3p in hippocampus promoted inflammatory response. **A** Immunofluorescent staining showed the Iba-1-positive microglia. **B** The number of Iba-1-positive microglia within the hippocampal region was significantly increased after knock-down of miR-26a-3p. **C** The microglia significantly activated in miR-26a-3p knock-down rats. **D** The expression of CD11b and CD45 was significantly increased in miR-26a-3p knock-down rats. **E**–**H** The pro-inflammatory cytokines were increased in miR-26a-3p knock-down rats. **I**, **J** The anti-inflammatory cytokines were increased in miR-26a-3p knock-down rats. Data are presented as the means ± SEMs. *N* = 6–8 per group. *P < 0.05, **P < 0.01, ***P < 0.001

Immunofluorescent staining revealed that the number of Iba-1-positive microglia within the hippocampal region was significantly increased after knock-down of miR-26a-3p (*P* < 0.01; Fig. 3A, B). The microglia significantly activated as presented by inflated cell soma and retraction of ramified processes (Fig. 3C). Consistently, the protein expression of CD11b and CD45 were increased after knock-down of miR-26a-3p (*P* < 0.01 for CD11b; *P* < 0.05 for CD45, Fig. 3D). Moreover, the mRNA expressions of several critical pro-inflammatory cytokines, such as interleukin-1β (IL-1β) (*P* < 0.05, Fig. 3E), IL-6 (*P* < 0.01, Fig. 3F), interferon gamma (IFN-γ) (*P* < 0.01, Fig. 3G) and tumor necrosis factor-α (TNF-α) (*P* < 0.001, Fig. 3H) within the hippocampal region were all significantly increased, whereas mRNA expressions of anti-inflammatory cytokines IL*-4 (*P < 0.001, Fig. 3I) and IL-10 (*P* < 0.01, Fig. 3J) were significantly decreased as compared to that observed in the control group. These results suggested that miR-26a-3p deficiency promoted inflammatory responses in hippocampus in rats.

### MiR-26a-3p deficiency caused dendrite spine loss in hippocampal neurons in rats

**Fig. 4:**
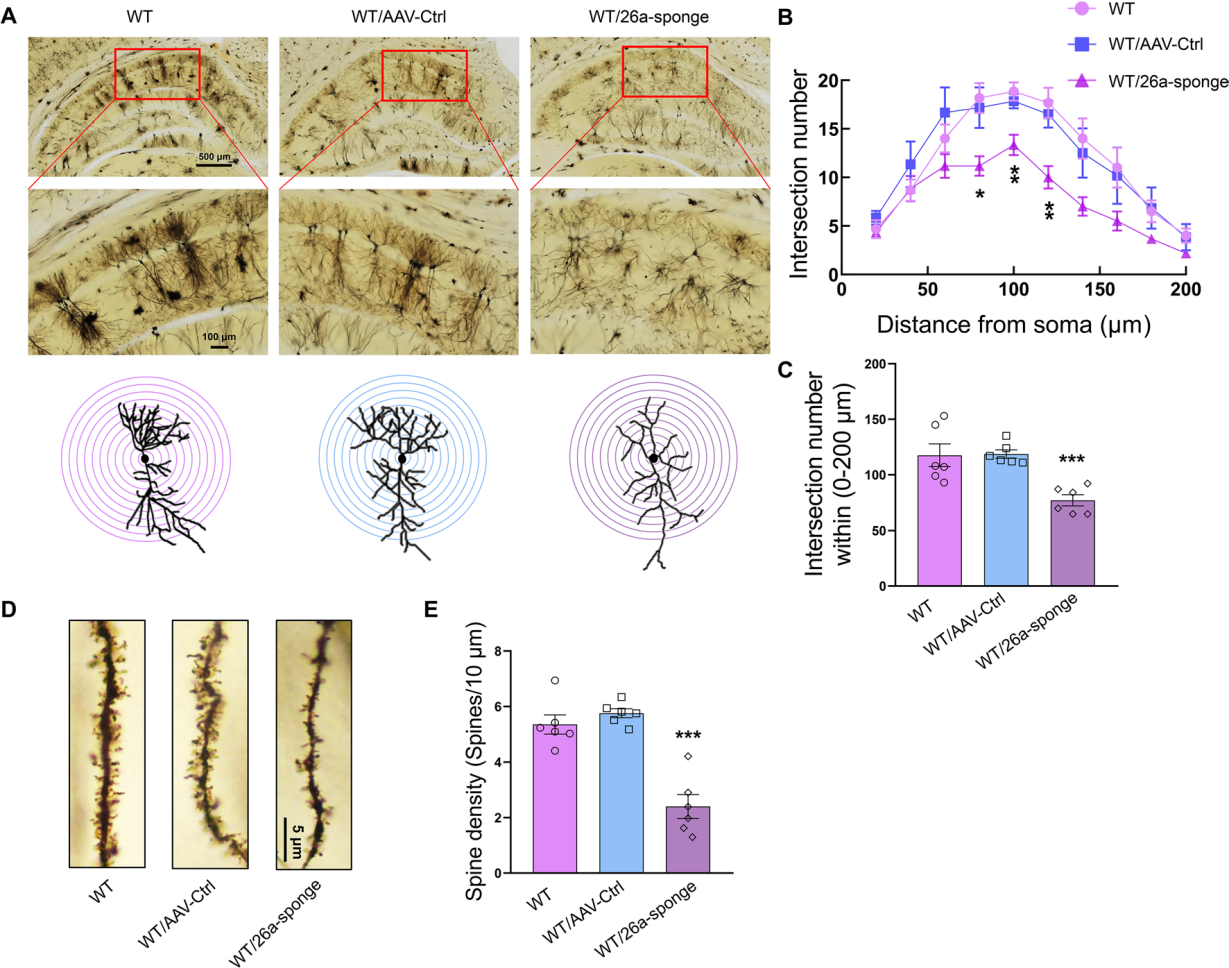
Knock-down miR-26a-3p caused dendrite spine loss in hippocampal neurons. **A** Representative images of hippocampus via Golgi staining. **B** The dendritic intersections against the radial distance from the soma were significantly decreased after knock-down miR-26a-3p. **C** The intersection number within 200 μm was decreased after knock-down of miR-26a-3p. **D** Representative images of dendrite in neurons of hippocampus. **E** The number of dendritic spines in hippocampal neurons was decreased after knock-down of miR-26a-3p. Data are presented as the means ± SEMs. *N* = 6 per group. 30 dendritic segments from 6 rats per group. *P < 0.05, **P < 0.01, ***P < 0.001

Then we investigated whether knock-down of miR-26a-3p result in dysregulation of synaptic plasticity in hippocampal neurons. Results from Golgi staining demonstrated that compared to the control group, the dendritic intersections against the radial distance from the soma of pyramidal neurons (*P* < 0.05, Fig. 4A, B) and the intersection number within 200 μm (*P* < 0.001, Fig. 4C) were significantly decreased, accompanied with the noticeable dendritic spine loss after knock-down of miR-26a-3p in hippocampal neurons (*P* < 0.001, Fig. 4D, E). These results revealed that the enhanced inflammatory response induced by miR-26a-3p deficiency maybe caused by a deterioration of neuronal structure, which may be associated with the synaptic transmission and depression-like and anxiety-like behaviors observed in these rats.

### MiR-26a-3p deficiency caused synaptic dysfunction in hippocampal neuron in rats

**Fig. 5:**
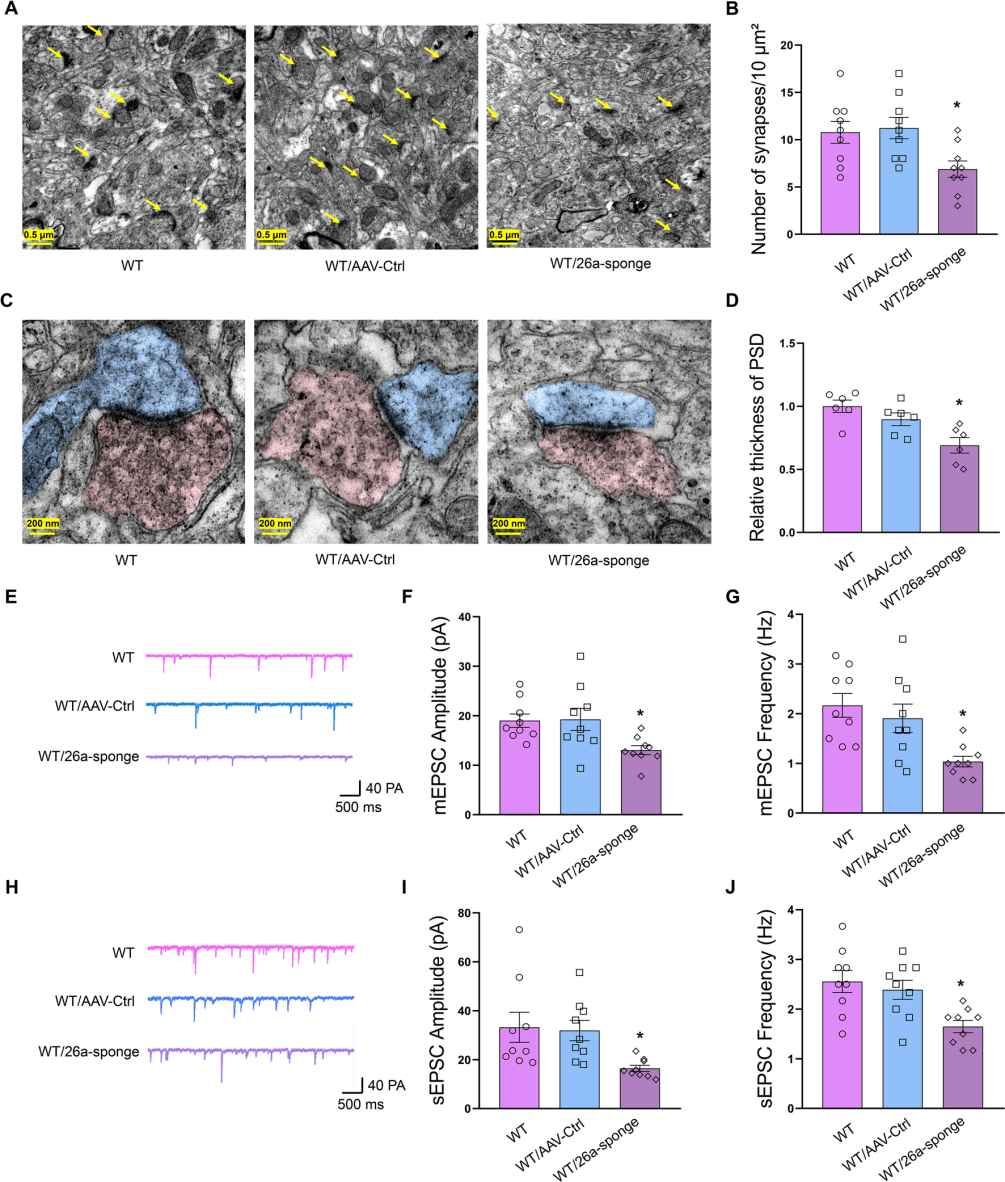
Knock-down miR-26a-3p caused synaptic dysfunction in hippocampal neurons. **A** Representative electronic micrographs of hippocampal neuron. **B** Knock-down miR-26a-3p decreased the number of synapses in hippocampal neuron. **C** Representative images of the ultrastructure of synapse. **D** Knock-down miR-26a-3p decreased in the thickness of post-synaptic density zone of synapse. **E** Raw traces of mEPSC by patch-clamp recordings. **F** Knock-down miR-26a-3p decreased the amplitude of mEPSC. **G** Knock-down miR-26a-3p decreased the frequency of mEPSC. **H** Raw traces of sEPSC by patch-clamp recordings. **I** Knock-down miR-26a-3p decreased the amplitude of sEPSC. **J** Knock-down miR-26a-3p decreased the frequency of sEPSC. Data are presented as the means ± SEMs. *N* = 9 neuros from 4 rats per group. *P < 0.05, **P < 0.01, ***P < 0.001

TEM analysis was used to observe the ultrastructure of hippocampal neurons. Representative electronic micrographs showed that the number of synapses within hippocampal neurons of was significantly reduced in miR-26a-3p knock-down rats as compared to the control group (*P* < 0.05, Fig. 5A, B). Consistently, the thickness of the post-synaptic density (PSD) was significantly reduced after knock-down of miR-26a-3p (*P* < 0.05, Fig. 5C, D). Moreover, whole-cell patch-clamp recordings demonstrated that these structural synaptic abnormities caused by miR-26a-3p knock-down were subsequently caused functional changes in excitatory pyramidal neurons. The frequency and amplitude of miniature and spontaneous excitatory post-synaptic currents (EPSCs) of hippocampal pyramidal neurons were significantly decreased in miR-26a-3p knock-down rats (*P* < 0.05, Fig. 5E–J). These results suggest that miR-26a-3p deficiency suppressed the efficiency of synaptic transmission in pyramidal neurons, which may then contribute to the behavioral disorders in these rats.

### The p38 MAPK mediated the miR-26a-3p deficiency-induced behavior disorders in rats

**Fig. 6:**
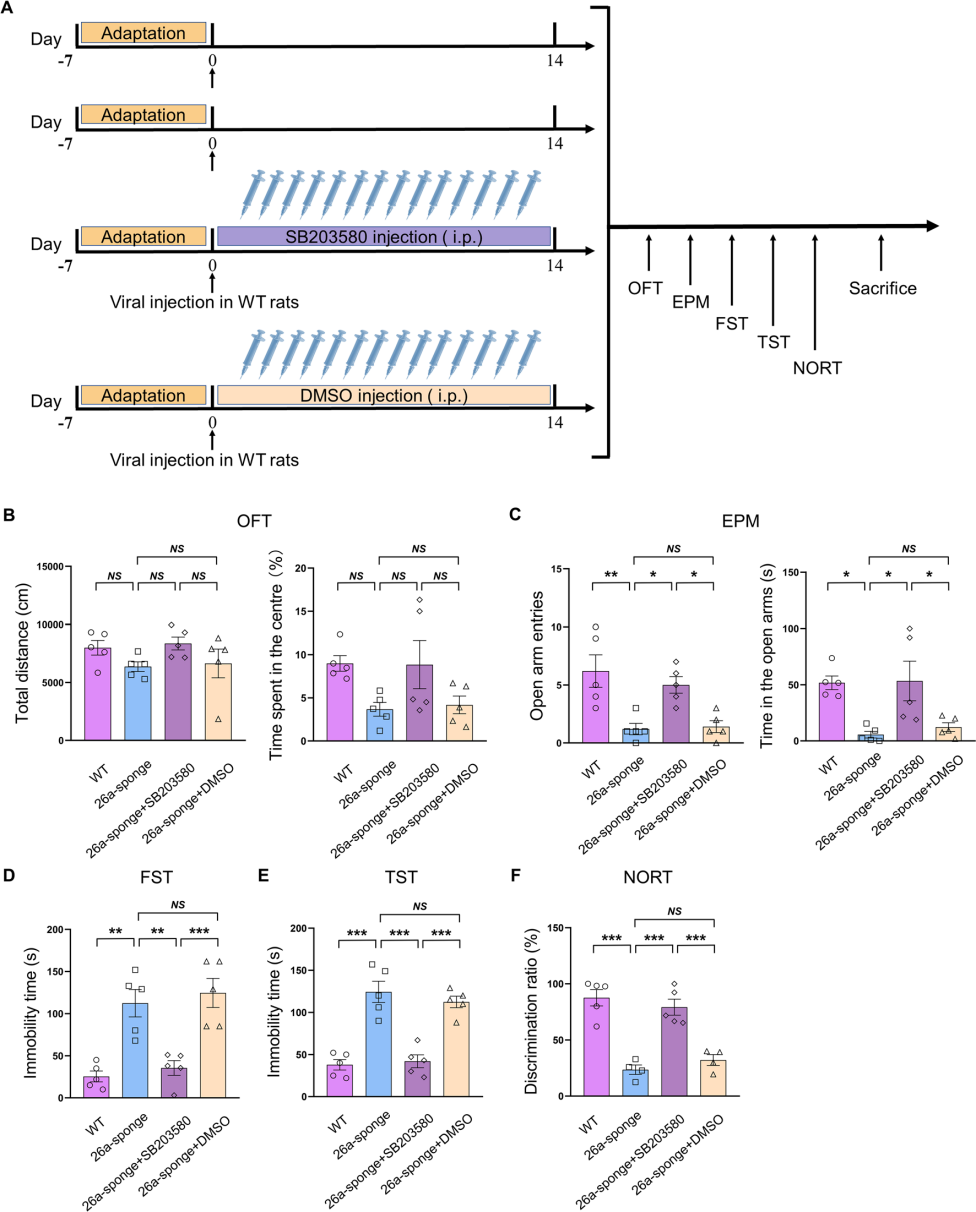
The p38 MAPK mediated the miR-26a-3p deficiency-induced behavior disorders. **A** Experimental paradigm of rats injected AAV-miR-26a-3p sponge virus followed by administration of SB203580, a specific antagonist of p38 MAPK. **B** Behavioral responses of OFT in miR-26a-3p knock-down and SB203580-treated rats. **C** Behavioral responses of EPM in miR-26a-3p knock-down and SB203580-treated rats. **D** Behavioral responses of FST in each group. **E** Behavioral responses of tail suspension test (TST) in each group. **F** Behavioral responses of novel object recognition test (NORT) in each group

Then we used SB203580, a specific antagonist of p38 MAPK, to further verify whether p38 signaling pathway is responsible for the mechanisms underlying the neuroinflammation and dysregulation of neuroplasticity caused by miR-26a-3p deficiency (Fig. 6A). The behavioral results showed that daily treatment with SB203580 for 2 weeks significantly reversed the behavioral changes in miR-26a-3p deficiency rats. SB203580 treatment has no changes on the locomotion of miR-26a-3p deficiency rats in the open filed test (*P* > 0.05, Fig. 6B), but significantly reversed the anxiety-like behaviors (*P* < 0.05, Fig. 6C) and depression-like behaviors (*P* < 0.01, Fig. 6D; *P* < 0.001, Fig. 6E) caused by miR-26a-3p knock-down. More interestingly, SB203580 treatment ameliorated the memory impairment caused by miR-26a-3p knock-down in the novel object recognition test (NORT) (*P* < 0.001, Fig. 6F). These results suggested that inhibition of p38 pathway ameliorated behavioral disorders in miR-26a-3p deficiency rats.

### The p38/NF-κB/NLRP3 pathway is involved in the miR-26a-3p deficiency-induced neuronal dysfunction in rats

**Fig. 7:**
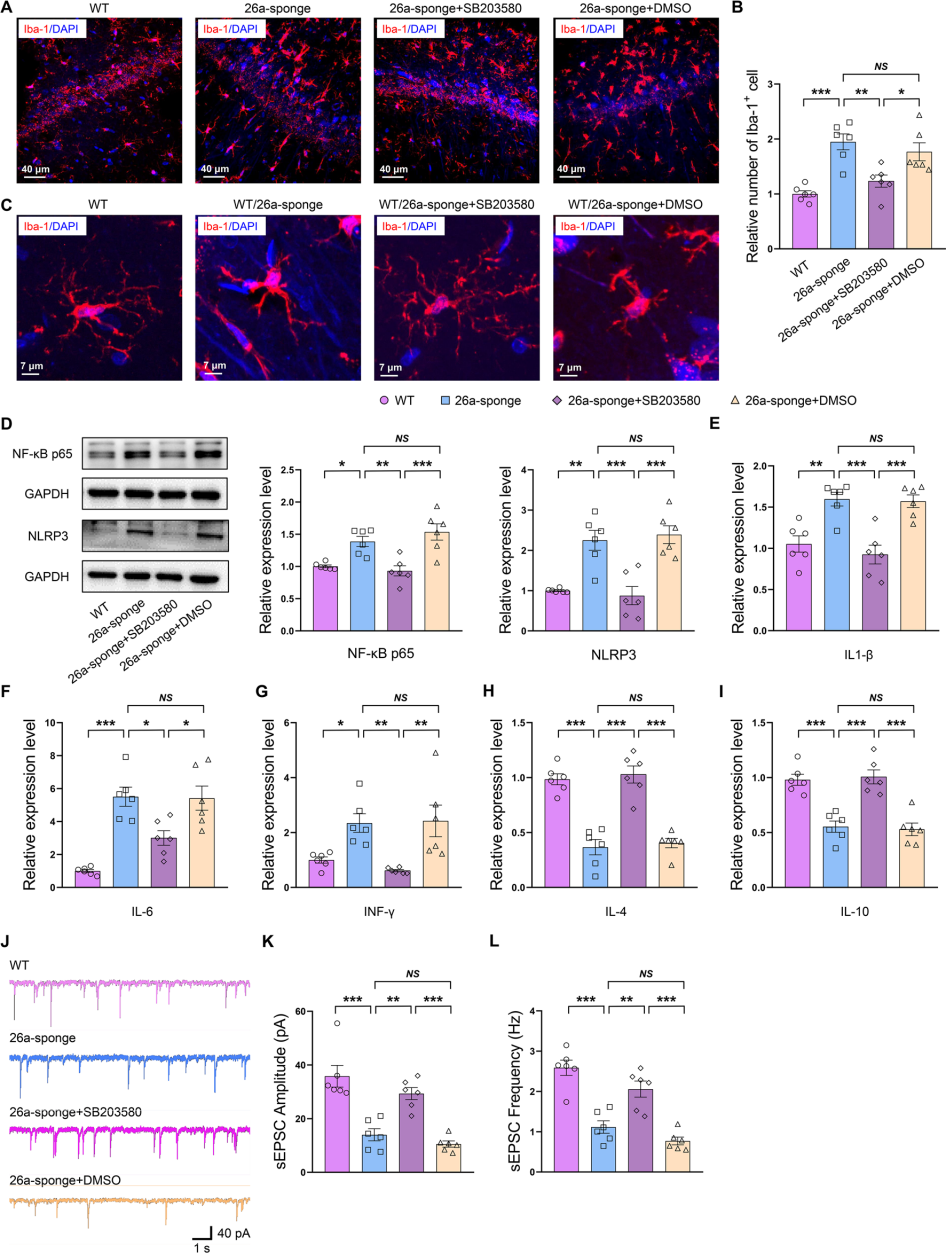
The p38/NF-κB/NLRP3 pathway mediated the miR-26a-3p deficiency-induced neuronal dysfunction. **A** Immunofluorescent staining showed Iba-1-positive microglia. **B** The number of Iba-1-positive microglia within the hippocampal region was decreased after daily treatment with SB203580 for 2 weeks in miR-26a-3p knock-down rats. **C** The microglia showed the resting states after the daily treatment with SB203580 for 2 weeks in miR-26a-3p deficiency rats. **D** SB203580 treatment reduced the increased phosphorylated level of p65 and expression level of NLRP3 induced by knock-down of miR-26a-3p. **E**–**G** SB203580 treatment reduced the increased level of IL-1β, IL-6 and IFN-γ induced by knock-down of miR-26a-3p. **H**, **I** SB203580 treatment has increased levels of IL-4 and IL-10 reduced by knock-down of miR-26a-3p. **J** Raw traces of sEPSC by patch-clamp recordings. **K** SB203580 treatment reversed the decreased amplitude of sEPSC. **L** SB203580 treatment reversed the decreased frequency of sEPSC. Data are presented as the means ± SEMs. *N* = 6–8 per group. For electrophysiological recordings, *N* = 6 neuros from 4 rats per group. *P < 0.05, **P < 0.01, ***P < 0.001

Moreover, immunofluorescent staining revealed that the number of Iba-1-positive microglia within the hippocampal region was decreased after daily treatment with SB203580 for 2 weeks in miR-26a-3p knock-down rats (*P* < 0.05, Fig. 7A, B). The microglia showed the resting states as presented by normal cell soma and ramified processes (Fig. 7C). The protein levels of the activated p65 NF-κB and NLRP3 (*P* < 0.001, Fig. 7D), as well as the mRNA levels of pro-inflammatory cytokines IL-1β (*P* < 0.05, Fig. 7E), IL-6 (*P* < 0.05, Fig. 7F) and IFN-γ (*P* < 0.05, Fig. 7G) which elevated by miR-26a-3p deficiency were also significantly suppressed by SB203580-treatment, while the mRNA expression levels of anti-inflammatory cytokines IL-4 (*P* < 0.01, Fig. 7H) and IL-10 (*P* < 0.01, Fig. 7I) was significantly increased. Results from electrophysiological recordings revealed that the decreased amplitude (*P* < 0.001, Fig. 7J, K) and frequency (*P* < 0.001, Fig. 7L) of sEPSC resulting from miR-26a-3p knock-down were also reversed by SB203580 injection. These results suggested that the p38/NF-κB/NLRP3 pathway acts as the target of miR-26a-3p, may contribute to the inflammatory response and involved in the synaptic dysfunction, while inhibition of p38 pathway may ameliorate these neuronal injury and behavioral disorders in miR-26a-3p deficiency rats via exerting neuroprotective effects.

## Discussion

Neuroinflammation is widespread in neuropsychiatric diseases and associated with neuronal injury within specific brain regions which induced behavioral disorders. The present study explored whether miR-26a-3p deficiency exerts neuronal injury effects via promoting inflammatory responses, in particular investigate the underlying mechanisms involved in the abnormal behaviors induced by miR-26a-3p deficiency. The adverse effects of miR-26a-3p deficiency appear to partially result from neural deterioration caused by neuroinflammation and apoptosis via upregulation of the p38 MAPK signaling pathway within the hippocampus, which provide some potential novel therapeutic targets in the treatment of inflammation-related neurological disorders.

The hippocampus, in particular the CA1 region, has recently been implicated as an important site involved in emotional and cognitive regulation [36, 37]. To the best of our knowledge, no studies that currently existed has focused on the miR-26a-3p deficiency as related to inflammation and synaptic transmission of neurons within the CA1 hippocampus. The mechanisms leading to the neural deterioration induced by miR-26a-3p deficiency and whether inhibiting this signaling pathway could be associated with the roles to exert neuroprotective effects are still unclear. The p38 MAPK family consists of highly conserved proline-directed serine-threonine protein kinases that are activated in response to environmental stresses including many growth factors, cytokines, and chemotactic substances [38]. It is well known that p38 MAPK is involved in the regulation of inflammation, apoptosis and cell differentiation. Previous results reported that p38 MAPK transduces signals from cell membranes to the nucleus to regulate gene expression for responses to environmental changes during stress-induced neuronal dysfunction, a process which is believed to underlie brain injury [39]. Bioinformatics database (TargetScan and MiRNAbase) predicted that the 3′-UTR of p38 mRNA would contain a putative binding site for the seed match sequence of miR-26a-3p, suggested that the p38 MAPK might be one of the target genes of miR-26a-3p. Therefore, mir-26a-3p may act as a sponge to p38 mRNA via complementary binding to the 3′-UTR region of p38 mRNA to inhibit the translation process of p38 protein, through which execute the regulation of neuronal function and behavioral performance. In this study, we showed that knock-down miR-26a-3p in hippocampus significantly increased the protein expression levels of p38 MAPK. These findings indicated that miR-26a-3p may exerts neuroprotective effects by targeting the regulation of P38 expression.

Increasing evidence indicates that p38 activity is critical for normal immune and inflammatory responses [40, 41]. It has been shown that p38 regulates the expression of multiple cytokines, transcription factors, and cell surface receptors, including cytosolic phospholipase A2, the microtubule-associated protein Tau, and the transcription factors ATF1 and -2, NF-κB and p53 [42]. In the present study, we further found that NF-κB, a transcription factor, was activated after knock-down of miR-26a-3p within hippocampus. NF-κB was reported can induce the priming of NLRP3 inflammasome, an important risk factor which was associated with the inflammation and cell death [43]. Here, we also found the level of NLRP3 was also increased after knock-down of miR-26a-3p. Therefore, p38 kinase may target NF-κB/NLRP3 signaling to lead to the enhanced inflammatory responses caused by miR-26a-3p deficiency. In recent years, much effort has been directed toward preventing glial activation and the release of pro-inflammatory cytokines as potential therapeutic approaches in the treatment of brain injury and thus behavioral disorders [44, 45]. In the present study, we found that miR-26a-3p deficiency in the hippocampal region induced anxiety-like and depression-like behaviors in rats, effects accompanied with an augment of glia activation which was represented as cellular hypertrophy of microglia. Moreover, miR-26a-3p deficiency also caused increased expressions of pro-inflammatory cytokines IL-1β, IL-6, interferon gamma (IFN-γ) and tumor necrosis factor-α (TNF-α), as well as caused reductions in anti-inflammatory cytokines IL-4 and IL-10 levels within hippocampal regions. Specifically, to determine whether the p38 MAPK pathway may contribute to the enhancement of neuroinflammation and thus the dysregulation of neuroplasticity in the injury effects of miR-26a-3p deficiency in rats, we injected SB203580, the antagonist of p38 MAPK, to block p38 pathway prior to knock-down of miR-26a-3p. The behavioral results showed that SB203580 pretreatment significantly ameliorate the depression-like and anxiety-like symptoms, which accompanied by decreased inflammatory response and enhanced synaptic transmission. Meanwhile, SB203580 also significantly reduced the activity levels of p65 NF-κB and the high expression levels of NLRP3, IL-1β, IL-6 and IFN-γ which induced by miR-26a-3p deficiency. Taken together, these results suggested that p38 MAPK signaling pathway may act as a downstream target of miR-26a-3p deficiency to contribute to the neuronal injury and behavioral disorders in rats.

Moreover, we further examined potential synaptic damage resulting from inflammation in this miR-26a-3p deficiency animal and found that pyramidal neurons within the hippocampus showed lowered number of spines, synapses and decreased post-synaptic density. More important, whole-cell patch-clamp recordings showed that these structural synaptic damages were accompanied with functional changes in pyramidal neurons. Knock-down of miR-26a-3p in hippocampus decreased the frequency and amplitude of miniature and spontaneous excitatory post-synaptic currents (EPSCs), suggest that this decreased synaptic transmission and excitability in pyramidal neurons may contribute to the anxiety-like and depression-like behaviors of these rats. These results suggest that the neuronal deterioration induced by miR-26a-3p deficiency within the hippocampus, as indicated by enhancement in the activation of microglia cells and the accumulation of inflammatory cytokines in this region, were accompanied by decreased number of dendrite spine and synapse resulting in reduction in synaptic transmission efficiency. These pathological characteristics of neuronal damage and the consequent synaptic dysfunction maybe the critical risk factors resulted in behavioral disorders in rats with miR-26a-3p deficiency. Taken together, these results suggest that miR-26a-3p deficiency may induce dysregulation of synaptic plasticity through neuronal inflammation, which was enhanced by activating the p38 MAPK signaling pathway.

While our current results reveal an important role for this miR-26a-3p/p38/NF-κB/NLRP3 pathway, it should be noted that neuropsychiatric disease is a complex disease involving potential interactions among gene–environment, neurotransmitter systems, neuronal injury and several other factors. Here, we focused on the effects of neuronal injury upon miR-26a-3p deficiency-induced behavioral and neurobiological changes in rats. We found that the upstream and downstream effects of p38 play an important role, but the detailed molecular and clinical mechanisms underlying the p38 signaling needs further investigation. Meanwhile, additional information regarding other mechanisms and details underlying the miR-26a deficiency will require further investigation.

## Conclusion

In conclusion, the findings of this study provide some new insights into the possible molecular mechanisms for the neuroprotective effects of miR-26a-3p. We demonstrate here that the deficiency of miR-26a-3p appears to, at least in part, caused dysregulation of synaptic plasticity that involved in the pathogenesis of behavioral disorders via activating its downstream target p38 MAPK signaling and thus promoting neuronal deterioration in rats. These results revealed that miR-26a-3p, which was enriched in central nervous system, functions as a critical regulator during neuronal steady state and may act as a potential therapeutic target in neurological disorders. Taken together, these findings suggest that targeting the inhibition of neuroinflammation and the consequent neuroplasticity dysregulation might serve as a new therapeutic strategy for the treatment of neurological disorders.

## Supplementary Information

**Additional file 1: Table S1. **Primer sequences of target genes used for Reverse transcription PCR in this study

## Data Availability

The data that support the findings of this study are available from the corresponding author upon reasonable request.

## Abbreviations

AAV: Adenovirus associated virus
ANOVA: Analysis of variance
CUMS: Chronic unpredictable mild stress
DAPI, 4′: 6-Diamidino-2-phenylindole dihydrochloride
FST: Forced swim test
ELISA: Enzyme linked immunosorbent assay
EPM: Elevated plus maze
Iba-1: Ionized calcium binding adaptor molecule-1
IFN-γ: Interferon gamma
IL-1: Interleukin 1
i.p.: Intraperitoneal
OFT: Open field test
PBS: Phosphate buffer saline
PFA: Paraformaldehyde
P38 MAPK: P38 mitogen-activated protein kinase
RT-PCR: Reverse transcription PCR
SPT: Sucrose preference test
SSRI: Selective serotonin reuptake inhibitor
TNF-α: Tumor necrosis factor-α
