# Supplementary material for: Hippocampal microRNA-26a-3p deficit contributes to neuroinflammation and behavioral disorders via p38 MAPK signaling pathway in rats
Source: J Neuroinflammation. 2022 Nov 24;19:283. doi: 10.1186/s12974-022-02645-1 (PMC9694101; doi:10.1186/s12974-022-02645-1)
Supplement: Supplementary file 1 — Additional file 1: Table S1. Primer sequences of target genes used for Reverse transcription PCR in this study [file 12974_2022_2645_MOESM1_ESM.docx]

Table S1. Primer sequences of target genes used for Reverse transcription PCR in this study

| Gene | Forword (5’→3’) | Reverse (5’→3’) |
| --- | --- | --- |
| IL-1β | AAGATGAAGGGCTGCTTCCAAACC | ATACTGCCTGCCTGAAGCTCTTGT |
| IFN-γ | ATTCATGAGCATCGCCAAGTTC | TGACAGCTGGTGAATCACTCTGAT |
| TNF-α | TGATCGGTCCCAACAAGGA | TGCTTGGTGGTTTGCTACGA |
| IL-6 | TGATCGGTCCCAACAAGGA | CCACTTGTTGGCTTATGTT |
| IL-10 | GCTCAGCACTGCTATGTTGC | TTGTCACCCCGGATGGAATG |
| IL-4 | CGTGATGTACCTCCGTGCTT | ATTCACGGTGCAGCTTCTCA |
| GAPDH | TCTCTGCTCCTCCCTGTTC | ACACCGACCTTCACCATCT |
